# Optimal adjuvant therapy for resected hepatocellular carcinoma: a systematic review with network meta-analysis

**DOI:** 10.18632/oncotarget.4098

**Published:** 2015-06-08

**Authors:** Gui-Qi Zhu, Ke-Qing Shi, Hua-Jian Yu, Sun-Yue He, Martin Braddock, Meng-Tao Zhou, Yong-Ping Chen, Ming-Hua Zheng

**Affiliations:** ^1^ Department of Infection and Liver Diseases, Liver Research Center, The First Affiliated Hospital of Wenzhou Medical University, Wenzhou, China; ^2^ School of the First Clinical Medical Sciences, Wenzhou Medical University, Wenzhou, China; ^3^ Institute of Hepatology, Wenzhou Medical University, Wenzhou, China; ^4^ Global Medicines Development, AstraZeneca R&D, Loughborough, United Kingdom; ^5^ Department of Hepatobiliary Surgery, The First Affiliated Hospital of Wenzhou Medical University, Wenzhou, China

**Keywords:** hepatocellular carcinoma, adjuvant therapy, toxic effect, network meta-analysis, indirect comparison

## Abstract

**Objectives:**

Major adjuvant therapies (ATs) for resected hepatocellular carcinoma (HCC) include chemotherapy, internal radiation therapy (IRT), interferon therapy (IFNT) and immunotherapy but the optimum regimen remains inconclusive. We aim to compare these therapies in terms of patient survival and recurrence rates.

**Methods:**

We searched PubMed, EMBASE and Cochrane library databases for randomized trials comparing the above four therapies until 31 March 2014. We estimated the HRs for survival and ORs for overall recurrence among different therapies. Toxic effects were also evaluated.

**Results:**

Fourteen eligible articles were included. IFNT improved 5-year survival greatly (HR 1.81, 95% CI 1.01–3.81, *P* = 0.034), whereas chemotherapy (HR 0.33, 95% CI 0.03–2.02), IRT (HR 0.31, 95% CI 0.02–3.33) and immunotherapy (HR 0.73, 95% CI 0.05–9.12) all provided a poorer survival outcome after 1-year. Similarly, for 5-year survival rates, although differing, IRT did not provide a significant improvement in survival (HR 1.38, 95% CI 0.34–5.19) compared with IFNT. Chemotherapy (HR 0.49, 95% CI 0.18–1.14) and immunotherapy (HR 0.56, 95% CI 0.17–1.59) did not appear to provide benefit over IFNT. Chemotherapy was ranked the worst in overall recurrence (OR 0.99, 95% CI 0.18–5.38) and most likely to cause toxic effects.

**Conclusions:**

IFNT was the most efficacious AT regimen both for short and long term survivals. Immunotherapy and IFNT were the most two effective in preventing overall relapse for resected HCC.

## INTRODUCTION

Hepatocellular carcinoma (HCC), the most common type of hepatobiliary cancer, ranks sixth among malignant tumors in incidence and is the third leading cause of cancer-related death [1]. The global incidence of HCC has continuously increased, with Asian countries accounting for almost 80% of victims worldwide [2–4]. Of the therapies aimed at cure, liver resection remains the optimal choice. Unfortunately, the recurrence rate of HCC 3 years after pure surgical resection is more than 50%, which is also the main cause of death after treatment [5]. Adjuvant therapy (AT) has been advocated to reduce relapse and prolong survival after surgery. Several adjuvant modalities have been developed in the past decades, nevertheless, the clinical use of these therapies remains controversial [6].

The results of previous studies involving interferon therapy (IFNT) showed that there was significant benefit after curative resection of HCC in terms of both survival and tumor recurrence [7–8]. Consistently, postoperative immunotherapy (IMT) and internal radiation therapy (IRT) might prevent recurrence after radical resection of HCC. However, IMT was found not to improve overall survival [9]. The role of chemotherapy (CT) was evaluated in several studies. Adjuvant CT with uracil-tegafur (UFT) after surgery in HCC patients suggested that it might worsen overall survival [10–12]. However, another study had showed the opposite result; that CT might postpone the recurrence of HCC and was likely to improve postoperative survival [13]. Several previous traditional meta-analyses [14–16] reported that IFNT might be a promising choice for the resection of HCC and also showed encouraging results with IMT [17]. With a lack of a direct comparison between ATs and observation alone for patients with surgically resected HCC, the question of which AT is optimal for the patient still remains inconclusive.

Opinions differ concerning a definition of optimum AT for resected HCC, which theoretically may be answered by conducting a very large clinical trial with multiple comparator arms. However, due to a lack of head-to-head trials making direct comparisons of certain treatments impossible, and the unfeasibility of estimating the effect for regimen comparison of more than two treatments at the same time, performing traditional meta-analysis is a challenging task. Bayesian network meta-analysis, also known as mixed treatment comparison, is a potential solution to the above problems. Mixed treatment comparison enables indirect comparison using a common comparator when a head-to-head trial is not available and combines direct and indirect comparisons to simultaneously compare several treatments [18–20].

To establish the optimum AT for HCC, we performed a random-effects network meta-analysis to compare the efficacy of major ATs (CT, IFNT, IRT or IMT) in terms of 1-year, 5-year survival and overall recurrence rates, and also evaluated the toxic effects of these ATs after surgical resection for HCC.

## RESULTS

### Study characteristics

**Figure 1:**
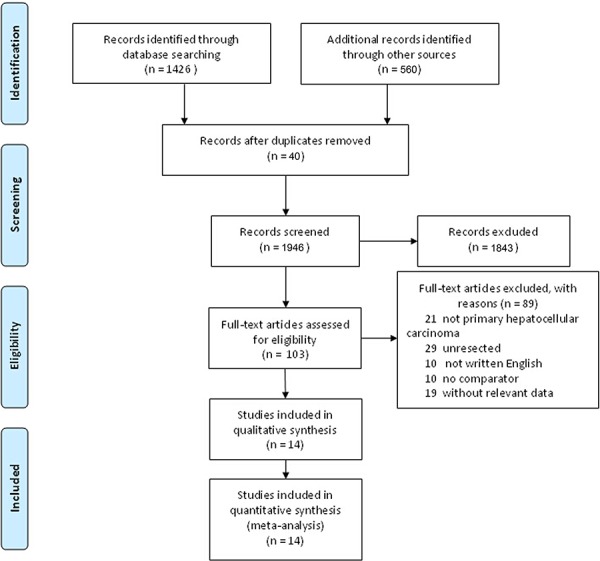
Study selection

**Figure 2:**
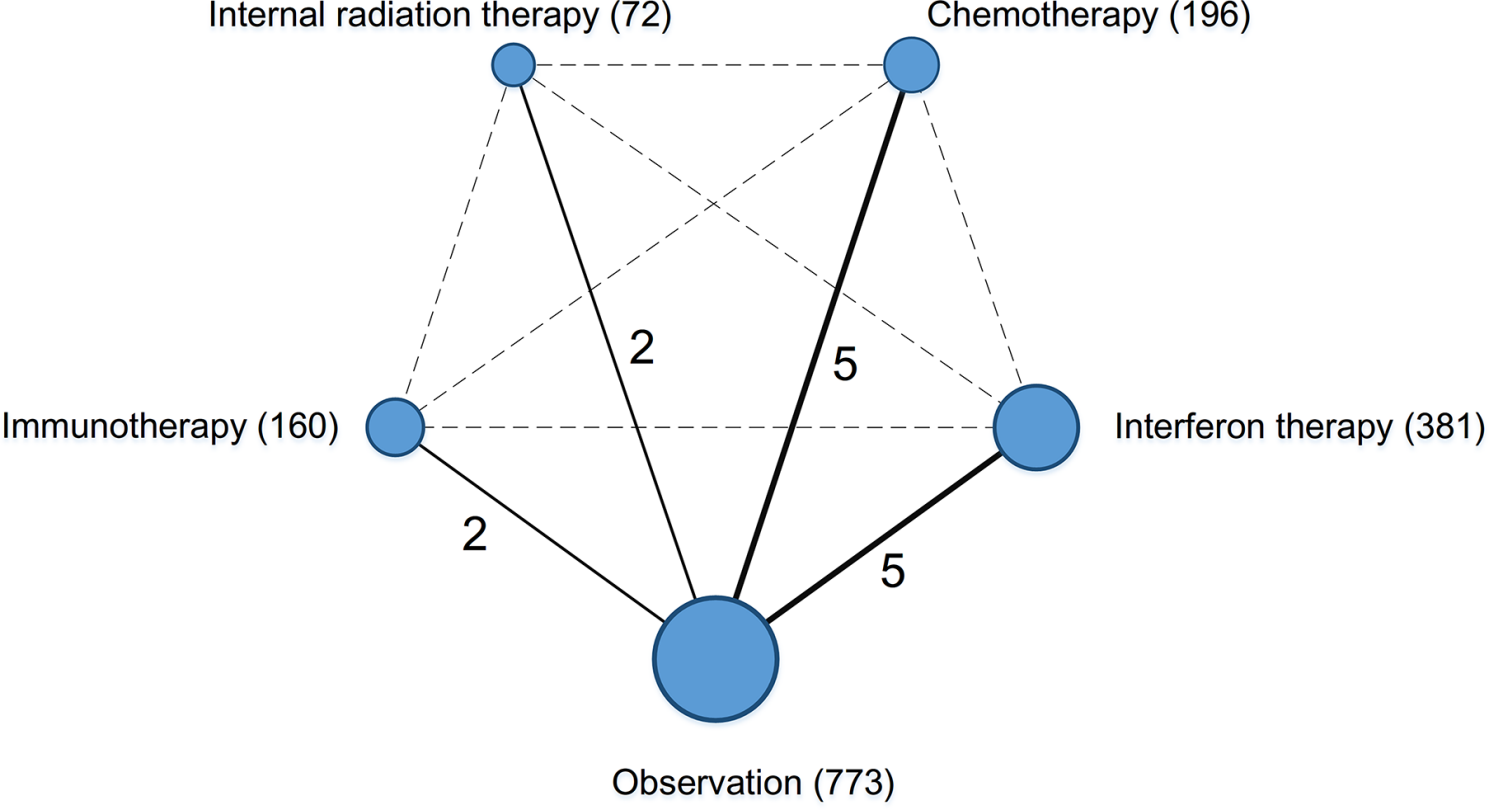
Network of the comparisons for the Bayesian network meta-analysis The size of the nodes is proportional to the number of patients (in parentheses) to receive the treatment. The width of the lines is proportional to the number of trials (beside the line) comparing the connected treatments.

**Figure 3:**
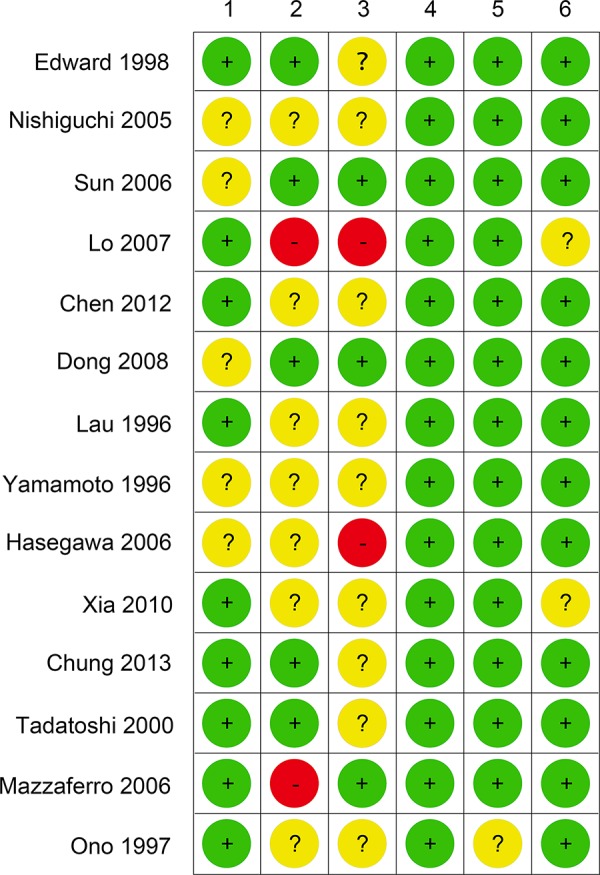
Cochrane risk of bias tool results 1: adequate sequence generation; 2: allocation concealment; 3: blinding; 4: incomplete outcome data address; 5: free of selective reporting; 6: free of other source of bias.

We identified 1986 studies for review by title and abstract (Fig. 1). After initial screening, we retrieved the full text of potentially eligible articles for detailed assessment, 1972 studies were excluded. Fourteen eligible studies were included for meta-analysis, with a total of 1582 patients who received one of the five treatment strategies (Fig. 2). Studies involved different counties, five studies [7, 10, 12, 21–22] were reported in Japan, six [8–9, 13, 23–25] from China and three [11, 26–27] are from Singapore, Italy and the United States, respectively. In terms of study sample sizes, the number of patients ranged from 30 to 268. 773 patients were treated with surgery alone, and 809 received ATs. Among the fourteen studies, patients were treated with CT alone in five studies [10–13, 21], IFNT alone in five studies [7–8, 23, 25, 27], both IRT and IMT only in two studies [9, 22, 24, 26]. Table 1 summarized the characteristics of the 14 studies that met our inclusion criteria. Double blinding was not described among 14 studies, but single blinding was in 2 studies, 2 studies were open-label studies, and 11 studies did not report blinding. As assessed by the Cochrane Risk of Bias tool, inadequate blinding provided the largest risk of bias followed by inadequate allocation concealment (Fig. 3).

**Table 1:** Characteristics of included studies

Author (Year)	Country	Comparison	No. of Patients	1-Year Survival (%)	5-Year Survival (%)	Overall Recurrence (%)
			Treatment/Control	Treatment/Control	Treatment/Control	Treatment/Control
Edward (1998) (11)	United States	CT vs OBS	30/36	77/94	53/64	77/47
Nishiguchi (2005) (7)	Japan	IFT vs OBS	15/15	100/93	80/40	60/87
Sun (2006) (8)	Mainland, China	IFT vs OBS	118/118	90/75	60/45	57/60
Lo (2007) (32)	HongKong, China	IFT vs OBS	40/40	100/85	10/8	55/53
Chen (2012) (30)	Taiwan, China	IFT vs OBS	133/135	96/96	54/53	59/56
Dong (2008) (9)	China	IMT vs OBS	84/43	87/86	39/37	32/70
Lau (1996) (31)	HongKong, China	IRT vs OBS	21/22	95/95	10/5	29/59
Yamamoto (1996) (28)	Japan	CT vs OBS	28/27	93/81	39/33	43/48
Hasegawa (2006) (10)	Japan	CT vs OBS	79/80	100/100	44/50	73/71
Xia (2010) (13)	China	CT vs OBS	30/30	87/83	63/40	53/77
Chung (2013) (33)	Singapore	IRT vs OBS	51/52	86/90	47/42	37/48
Tadatoshi (2000) (29)	Singapore	IMT vs OBS	76/74	99/95	36/35	59/77
Mazzaferro (2006) (34)	Italy	IFT vs OBS	75/74	89/92	16/5	36/10
Ono (1997) (12)	Japan	CT vs OBS	29/27	93/96	31/56	66/70

NR = not reported; OBS = observation; IRT = internal radiation therapy; IFT = interferon therapy; CT = chemotherapy; IMT = immunotherapy.

### Efficacy of adjuvant therapy

**Figure 4:**
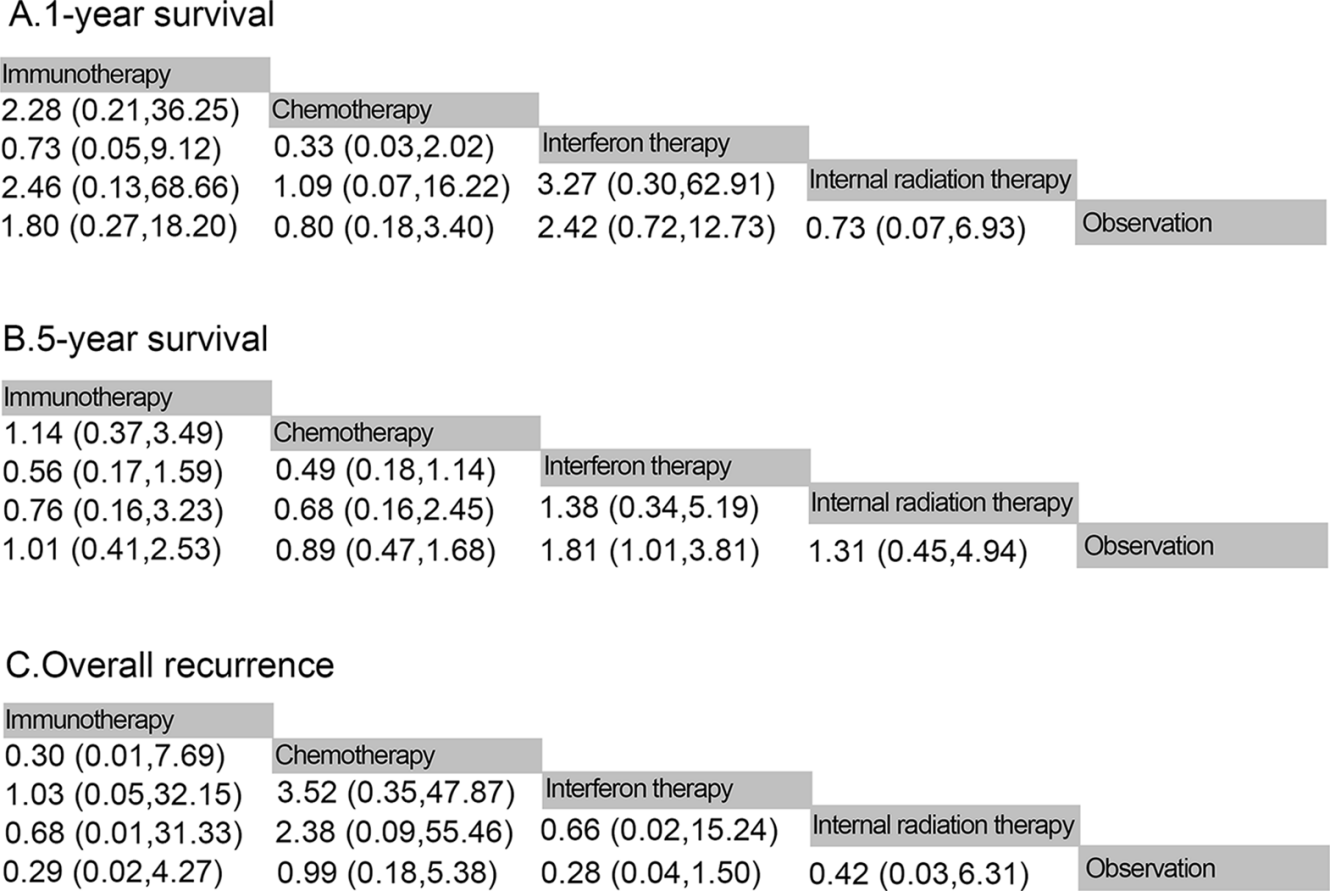
Pooled hazard ratios for death and pooled odds ratios for overall recurrence **A.** 1-year survival; **B.** 5-year survival; **C.** overall recurrencxe. The column treatment is compared with the row treatment. Numbers in parentheses indicate 95% credible intervals.

All fourteen trials reported information on 1-year, 5-year survival and overall recurrence rates. We summarized the results of the random-effects network meta-analysis for 1-year, 5-year survival and overall recurrence rates in Fig. 4. For 1-year survival (Fig. 4A), the difference is not statistically significant for all ATs, compared with observation, however, there is a tendency that IFNT improved 1-year survival rate (HR 2.42, 95% CI 0.72–12.73), whereas IMT (HR 0.73, 95% CI 0.05–9.12), IRT (HR 3.27, 95% CI 0.30–62.91) and CT (HR 0.33, 95% CI 0.03–2.02) provided a poorer 1-year survival rate compared with IFNT (Fig. 4A). For 5-year survival, Fig. 4B showed that when compared with observation, IFNT improved 5-year survival rates significantly (HR 1.81, 95% CI 1.01–3.81, *P* = 0.034). Although differing significantly, IRT did not provide a significant improvement in survival (HR 1.38, 95% CI 0.34–5.19) compared with IFNT. Both the CT (HR 0.49, 95% CI 0.18–1.14) and IMT (HR 0.56, 95% CI 0.17–1.59) did not provide benefit over IFNT. In terms of the overall recurrence rate (Fig. 4C), the results showed that CT was associated with higher recurrence rate than IMT (OR 0.30, 95% CI 0.01–7.69), IFNT (OR 3.52, 95% CI 0.25–47.87), IRT (OR 2.38, 95% CI 0.09–55.46) and observation (OR 0.99, 95% CI 0.18–5.38).

**Figure 5:**
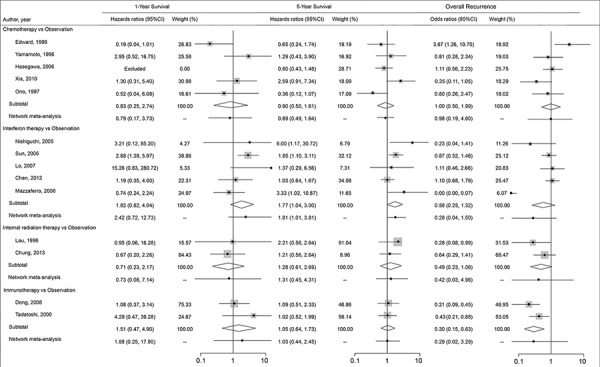
Pooled hazards ratios for death and pooled odds ratios for recurrence by Bayesian network meta-analysis and traditional meta-analysis

A direct comparison of results from traditional pair wise meta-analysis and network meta-analysis did not suggest inconsistency between direct and indirect evidence (Fig. 5).

**Figure 6:**
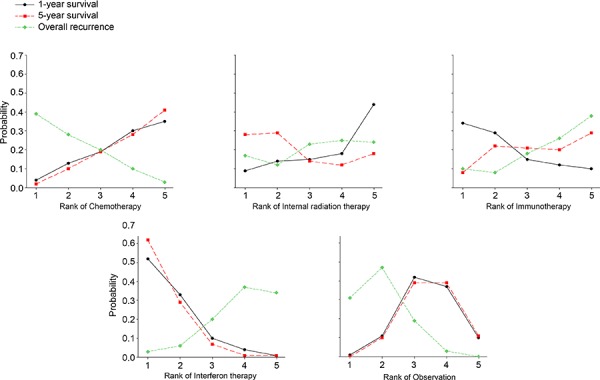
Ranking for death and recurrence of 5 interventions for resected hepatocellular carcinoma A: 1-year survival; B: 5-year survival; C: overall recurrence. Ranking indicates the probability to be the best treatment, the second best, the third best and so on. Rank 1 is worst and rank N is best.

The probabilities of best treatment for each intervention were ranked at each of the possible five positions (Fig. 6). IFNT and IMT had the highest probabilities of reduction in mortality rate for 1-year survival (Fig. 6A), suggesting IFNT and IMT were more efficacious than the other remaining interventions, the cumulative probabilities of being among the most efficacious interventions in improving the survival in the short term was IFNT. For 5-year survival, IFNT and IRT were more efficacious than the other remaining interventions (Fig. 6B), they had the highest probabilities of reduction in mortality rate in the long term, the cumulative probabilities of being among the most efficacious interventions in improving the survival in the long term was still IFNT. In terms of overall recurrence, Fig. 6C showed that IFNT and IMT had the highest probabilities of reduction in overall recurrence rate, suggesting that IFNT and IMT were also efficacious than the other interventions.

### Toxic effects

All trials reported data on side effects associated with each therapeutic regime. In the studies of CT [10–13, 21] and of IFNT [7–8, 23, 25, 27] 35% (69/196) and 32% (128/396) patients in adjuvant group, respectively, developed severe toxicity or treatment-related side effects requiring a discontinuation of AT, while IMT [9, 22] was associated with frequent but mild adverse effects. When compared with other adjuvant modalities, IRT [24, 26] appeared to be safe, because side effects of this therapy were rarely reported in the scientific literature.

## DISCUSSION

This network meta-analysis was based on fourteen studies including 1582 individuals comparing the major ATs, and also including both benefits and adverse events when comparing those treatments for resected HCC. Our results suggested that adjuvant IFNT provided an overall survival advantage over the remaining ATs and also offered a reduction of overall recurrence rate, but might increases toxic effects. Whereas CT failed to confer any benefit to overall survival and provided highest overall recurrence rate for resected HCC.

The meta-analysis showed that IFNT reduced long term mortality by about two times and postponed recurrence by about two thirds after resection of HCC. As previously reported, survival benefits and recurrence reduction is observed in all trials involving IFNT [7–8, 23, 25, 27–28], and two subsequent meta-analyses [14–15] concluded that the use of IFNT had a significant beneficial effect after resection of HCC, in terms of both survival and tumor recurrence. These observations are consistent with our results, although not differing significantly for 1-year survival and overall recurrence rates. A further meta-analysis in 2010 [29] concluded that IFNT, in particular, provided a significant beneficial effect when compared with observation alone (*P* < 0.05), using clinical data which were derived from indiscriminate pooling of various antiviral therapies as ATs. In addition, one recent meta-analysis in 2013 [30] showed that compared with OBS, adjuvant IFNT significantly improved the recurrence-free survival (HR 0.66, 95% CI 0.52–0.84) and overall survival (HR 0.43, 95% CI 0.34–0.56) of patients with viral hepatitis-related HCC following curative treatment. Consistently, another three traditional meta-analyses [16, 31] recently published also concluded that IFNT may significantly reduce the recurrence rates of patients with viral hepatitis-related HCC and improve the survival of patients after surgical resection. By contrast, our network meta-analysis which has the feature of dissection of the effectiveness of individual treatments, assessed IFNT, CT, IRT and IMT separately and progresses the field beyond conventional meta-analyses. Our results suggest that IFNT prolonged overall survival and postponed recurrence with a balanced benefit-toxicity ratio and that IFNT was the optimum treatment regimen after resection of HCC.

There was no further survival benefit and more frequent recurrence with CT, which were consistent with those of three previous trials [10–12]. A traditional meta-analysis showed that CT was not recommended due to the deterioration of the patient's long term outcome and frequent recurrence of the disease [32]. However, in another study [21], the use of CT seemed to have potential benefit on tumor recurrence, but it should be weighed against the risks of adverse reactions in patients. In a network meta-analysis, however, CT might not be the optimal choice according to the cumulative probabilities of being among the two least efficacious interventions for 1-year and 5-year survival outcomes. Our results also concluded that IMT cannot provide survival benefits but offer a lower recurrence, which were consistent with a previous traditional meta-analysis [17], according to the cumulative probabilities of being highest for recurrence and being the lowest for survival among interventions.

This study had some merits. This meta-analysis compared all major therapies simultaneously and assessed every therapy individually. We used the consistent measure of survival across different studies and synthesized all available studies within a single meta-analysis, avoiding potential selection bias [33]. Bayesian network meta-analysis also compared therapies indirectly when no head-to-head trial existed and get more precise effect estimates by assessing direct and indirect comparisons [19–20]. Furthermore, in order to obtain a favorable benefit-risk ratio for HCC by the major ATs, we analyzed the toxic effects and intended to determine what ATs was optimal for patients. This updated synthesis of existing evidence provided new insights into controversies on this issue with important implications in clinical care and future research.

Our findings do also have limitations. First, the quality of the included studies varied greatly. Randomization was adequate in all trials. However, information about allocation concealment and blinding were not adequately reported in most trials included in our analysis, which might undermine the validity of overall findings. However, the scant information in terms of quality assessment had been commonly found in other systematic reviews. Most studies included in this study were very similar in terms of design and conduct. Therefore, it could be more an issue of reporting in the text than real defects in study design. Second, the generalizability of the results was limited by predominantly including RCTs mainly conducted among Japanese and Chinese patients. Conceivably, there could be differences in the natural history of HCC among geographical regions, although these potential differences had not been well understood. Therefore, it is possible that the findings of the current meta-analysis might not be extrapolated to the non-Asian population. Third, the sizes were small in most studies included in this analysis. However, our study had established the largest sample size for trials on resected HCC performed to date in the world. Therefore, the results of this Bayesian meta-analysis might provide a beneficial and complete picture to support physicians in selecting ATs.

In summary, the network meta-analysis suggested that IFNT was the most efficacious AT regimen for both short and long term survivals. IMT could prevent recurrence but contribute less to survival benefits. There is no any benefit with CT to overall survival or recurrence, which was also associated with greater toxicity than any other ATs.

## MATERIALS AND METHODS

### Search strategy

The protocol for the systematic review was based on the PRISMA (Preferred Reporting Items for Systematic Reviews and Meta-Analyses) guideline (Supporting information 1) [34]. A systematic search of PubMed, Embase and Cochrane library databases was conducted using the key terms ‘hepatocellular carcinoma and adjuvant treatment’ until the end of March 2015 without language or date restrictions. A manual search was also performed of reference lists of published articles and literature searches were complemented by perusing the reference lists of previous meta-analyses.

### Selection criteria

In order to be included, a study had to fulfill the following criteria: (i) randomized controlled trials published as abstracts, letters to the editor or peer-reviewed articles; (ii) patients with HCC who had undergone potentially curative treatment with surgical resection; (ii) interventions: treatment with IFNT, IRT, CT or IMT; treatments for patients administered after curative-intent surgery; (iii) use of 1-year and 5-year survival rates or cumulative probability of overall recurrence as clinical end-points. Trials that enrolled the patients with metastatic hepatocellular carcinoma were excluded. Other exclusions were trials that comprised a non-randomized design, studies evaluating ATs after non-curative resection, and trials comparing other ATs.

### Data extraction

Two investigators (Zhu GQ, Shi KQ) independently reviewed the full manuscripts of eligible studies and extracted information into an electronic database, including publication data (the first author's name, year of publication, and country of population studied), treatment protocols that were compared and number of patients assigned to each group, the number of events of interest in each group, and outcomes (1-year, 5-year survival rates, overall recurrence rate). Any discrepancies regarding the extraction of data were resolved by additional investigator (Zheng MH). When needed data was not reported in the text, it was independently calculated from survival curves. Missing or not found data from studies deemed eligible were sought from the authors via e-mail request.

### Risk of bias

The quality of the methodology was independently assessed by two reviewers using the Cochrane Risk of Bias Tool, an established tool based on assessing sequence generation for the randomization of subjects, allocation concealment of treatment, blinding, incomplete outcome data, selective outcome reporting and other sources of bias. [35] Trials with high or unclear risk for bias for any one of the first three components were regarded as trials with high risk of bias. Otherwise, they were considered as trials with low risk of bias.

### Data analysis

First, we performed a traditional pair-wise meta-analysis which could synthesize studies that compared the same interventions with STATA 12.0 (Stata Corporation, College Station, Texas, USA). To account for heterogeneity between studies, a random-effects model for meta-analysis was utilized. The heterogeneity was assessed with the *I^2^* statistic, *a* value of more than 50%, was considered to be representative of statistically significant heterogeneity [36].

The relative frequency of survival at 1 year and 5 years between AT and non-AT (defined as observation) was expressed as an HR, which was the preferred outcome measured as HRs account for censoring and provide time-to-event information [33]. When HRs were not reported, they were estimated from summary statistics with the method described by Tierney et al [37]. Data were extracted from the primary publications and combined into a meta-analysis.

The pooled HRs from the network meta-analysis were compared with corresponding HRs from pair-wise random-effects meta-analysis of direct comparisons to assess whether there was inconsistency between direct and indirect comparisons. For overall recurrence, odds ratios (ORs) were calculated from the number of total patients and the number of patients in each trial for network meta-analysis.

In addition to the direct comparison meta-analyses, we also performed multiple-treatment meta-analyses with a random-effects model within a Bayesian framework using Markov chain Monte Carlo methods in WinBUGS (MRC Bio-statistics Unit, Cambridge, UK). Non-informative uniform and normal prior distributions were used and three various sets of starting values to fit the model, yielding 150 000 iterations (50 000 per chain) generating the posterior distributions of model parameters [19–20]. This method combined direct and indirect evidence for any given pair of treatments in one joint analysis [38–39]. In addition to analysis of the direct within-trial comparisons between two treatments, the network framework enabled the incorporation of indirect comparisons constructed from two trials that have one treatment in common. Detailed description of methods may be found in our previous network meta-analyses. [40–43] Treatments were ranked for each outcome in each simulation on the basis of their posterior probabilities. We assessed the probability that each adjuvant treatment was the most efficacious regimen, the second best, the third best and so on, by calculating the HR for each treatment compared with an arbitrary common group and counting the proportion of iterations of the Markov chain in which each treatment had the highest HR, the second highest, and so on. Therefore, the multiple-treatments meta-analysis increased statistical power by incorporating evidence from both direct and indirect comparisons across all interventions.
